# Whole-Genome Sequence of *Mycobacterium intracellulare* Clinical Strain MOTT-H4Y, Belonging to INT5 Genotype

**DOI:** 10.1128/genomeA.00006-13

**Published:** 2013-02-28

**Authors:** Hyungki Lee, Byoung-Jun Kim, Kijeong Kim, Seok-Hyun Hong, Yoon-Hoh Kook, Bum-Joon Kim

**Affiliations:** a Department of Microbiology and Immunology, Biomedical Sciences, Liver Research Institute, Institute of Endemic Diseases, Seoul National University, Medical Research Center (SNUMRC), Seoul National University, College of Medicine, Seoul, Republic of Korea; b Department of Microbiology, College of Medicine, Chung-Ang University, Seoul, Republic of Korea

## Abstract

Here, we report the draft genome sequence of the *Mycobacterium intracellulare* clinical strain MOTT-H4Y, grouped previously into the INT5 genotype of the 5 genotypes of *M. intracellulare*.

## GENOME ANNOUNCEMENT

Of members of the *Mycobacterium avium* complex (MAC), *Mycobacterium intracellulare* has been reported to be isolated more frequently than is *M. avium* in Korea (1–3). Previously, we reported that the 94 *M. intracellulare* clinical isolates from Korean patients were divided into 5 genotypes (INT1, INT2, INT3, INT4, and INT5) (4). Recently, we introduced the complete genome sequences of four *M. intracellulare* strains: two INT2 strains (ATCC 13950^T^ [GenBank accession no. CP003322] [5] and MOTT-02 [GenBank accession no. CP003323] [6]), one INT1 strain (MOTT-64 [GenBank accession no. CP003324] [7]), and one INT5 strain (MOTT-36Y [GenBank accession no. CP003491] [8]). To understand the phylogenetic and genetic backgrounds of INT5 strains showing phylogenetic distinctness from other *M. intracellulare* genotypes, whole-genome sequencing of another *M. intracellulare* INT5 clinical strain, MOTT-H4Y, was performed in this study.

The *Mycobacterium* sp. MOTT-H4Y genome was sequenced by a standard shotgun strategy using GS FLX pyrosequencing technology. Sequencing analysis was performed in the National Instrumentation Center for Environmental Management (NICEM) (genome analysis unit) at Seoul National University. A total of 787,165 reads were generated, with an average read length of 429, yielding 337,397,625 bp of the total sequences. This represents ~62× coverage for the estimated 5.4 Mb genome size. The assembled sequences contained three contigs (3,099,687 bp, 1,499,525 bp, and 819,111 bp) with a G+C content of 68.09% and a plasmid sequence (24,702 bp) with a G+C content of 65.4%. The obtained contigs were compared for mapping to the whole-genome sequences of the reference strains using the BLASTZ program (http://www.bx.psu.edu/miller_lab/). All the remaining gaps between contigs were completely filled by ~50-fold Solexa reads and PCR amplifications. Genome annotation was performed using the NCBI Prokaryotic Genomes Automatic Annotation Pipeline (PGAAP) (http://www.ncbi.nlm.nih.gov/genomes/static/Pipeline.html).

A comparison of the *Mycobacterium* sp. MOTT-H4Y genome with the *M. intracellulare* ATCC 13950^T^ and *Mycobacterium* sp. MOTT-36Y genomes (5, 8) reveals it to have a circular DNA of 5,418,323 bp with a plasmid of 24,702 bp. The genome of *Mycobacterium* sp. MOTT-H4Y contains similar numbers of protein-coding genes (5,233 open reading frames [ORFs]) as *M. intracellulare* ATCC 13950^T^ (5,145 ORFs) and *Mycobacterium* sp. MOTT-36Y (5,381 ORFs); however, the number of tRNA genes (48 tRNA genes) was greater than those of *M. intracellulare* ATCC 13950^T^ (47 tRNA genes) and *Mycobacterium* sp. MOTT-36Y (46 tRNA genes). The genome of *Mycobacterium* sp. MOTT-H4Y has a G+C content of 68.09%, and a plasmid was found in its genome with a G+C content of 65.4%. A comparison of predicted ORFs of *Mycobacterium* sp. MOTT-H4Y with *M. intracellulare* ATCC 13950^T^ and *Mycobacterium* sp. MOTT-36Y showed that they shared 4,685 ORFs (average identity, 95.9%) and 4,988 ORFs (average identity, 98.1%), respectively. Five hundred one ORFs (9.7%) and 547 ORFs (10.5%) were specific to *M. intracellulare* ATCC 13950^T^ and *Mycobacterium* sp. MOTT-H4Y, respectively, and 326 ORFs (6.1%) and 244 ORFs (4.7%) were specific to *Mycobacterium* sp. MOTT-36Y and *Mycobacterium* sp. MOTT-H4Y, respectively.

### Nucleotide sequence accession number. 

Nucleotide sequences of the chromosome and plasmid of *Mycobacterium* sp. MOTT-H4Y have been deposited in GenBank under the accession no. AKIG00000000.

## References

[1] Koh WJ Kwon OJ Jeon K Kim TS Lee KS Park YK Bai GH 2006 Clinical significance of nontuberculous mycobacteria isolated from respiratory specimens in Korea. Chest 129:341–348 16478850 10.1378/chest.129.2.341

[2] Koh WJ Kwon OJ Lee KS 2005 Diagnosis and treatment of nontuberculous mycobacterial pulmonary diseases: a Korean perspective. J. Korean Med. Sci. 20:913–925 16361797 10.3346/jkms.2005.20.6.913 PMC2779319

[3] Ryoo SW Shin S Shim MS Park YS Lew WJ Park SN Park YK Kang S 2008 Spread of nontuberculous mycobacteria from 1993 to 2006 in Koreans. J. Clin. Lab. Anal. 22:415–420 19021271 10.1002/jcla.20278 PMC6649107

[4] Park JH Shim TS Lee SA Lee H Lee IK Kim K Kook YH Kim BJ 2010 Molecular characterization of *Mycobacterium intracellulare*-related strains based on the sequence analysis of *hsp65*, internal transcribed spacer and 16S rRNA genes. J. Med. Microbiol. 59:1037–1043 20522624 10.1099/jmm.0.020727-0

[5] Kim BJ Choi BS Lim JS Choi IY Lee JH Chun J Kook YH Kim BJ 2012 Complete genome sequence of *Mycobacterium intracellulare* ATCC 13950^T^. J. Bacteriol. 194:2750 22535933 10.1128/JB.00295-12 PMC3347195

[6] Kim BJ Choi BS Lim JS Choi IY Lee JH Chun J Kook YH Kim BJ 2012 Complete genome sequence of *Mycobacterium intracellulare* clinical strain MOTT-02. J. Bacteriol. 194:2771 22535946 10.1128/JB.00365-12 PMC3347180

[7] Kim BJ Choi BS Lim JS Choi IY Kook YH Kim BJ 2012 Complete genome sequence of *Mycobacterium intracellulare* clinical strain MOTT-64, belonging to the INT1 genotype. J. Bacteriol. 194:3268 22628501 10.1128/JB.00471-12 PMC3370864

[8] Kim BJ Choi BS Choi IY Lee JH Chun J Hong SH Kook YH Kim BJ 2012 Complete genome sequence of *Mycobacterium intracellulare* clinical strain MOTT-36Y, belonging to the INT5 genotype. J. Bacteriol. 194:4141–4142 22815454 10.1128/JB.00752-12 PMC3416561

